# Household insecticide use and urinary 3-phenoxybenzoic acid levels in an elder population: a repeated measures data

**DOI:** 10.1038/s41370-020-00276-3

**Published:** 2020-10-06

**Authors:** Jin Hee Kim, Sungroul Kim, Yun-Chul Hong

**Affiliations:** 1 Department of Integrative Bioscience and Biotechnology, Sejong University, Seoul, 05006 Republic of Korea; 2 Department of Environmental Health Sciences, Soonchunhyang University, Asan, 336-745 Republic of Korea; 3 Institute of Environmental Medicine, Seoul National University Medical Research Center, Seoul, 110-799 Republic of Korea; 4 Department of Preventive Medicine, Seoul National University College of Medicine, Seoul, 110-799 Republic of Korea

**Keywords:** Pyrethroids, 3-Phenoxybenzoic acid, Elders, Insecticide spray

## Abstract

**Background:**

Pyrethroids are associated with adverse health consequences, even at low-dose exposures. However, there is limited evidence on pyrethroids exposure levels among vulnerable elder population and on their exposure sources.

**Objective:**

We tried to determine pyrethroids exposure levels among Korean elders and their exposure sources.

**Methods:**

We measured levels of 3-phenoxybenzoic acid (3-PBA), a pyrethroids metabolite, in urines repeatedly collected from 1239 Korean rural and urban elders; we also explored exposure sources for pyrethroids using questionnaire data.

**Results:**

Our participants had high levels of 3-PBA with 446 (36.0%) of elders with 3-PBA level over 2 ng/mL of 95th percentile of the German representative populations. After adjustment for sex, age, smoking status, visit episode, and surveyed season using linear mixed effect models, household insecticide spray use was significantly associated with 3-PBA level (*β* = 0.03 and *p* = 0.02) and the association was apparent only for females (*β* = 0.03 and *p* = 0.03). In the analyses for nonlinear relationships using generalized additive mixed models, there was a J-shape change in 3-PBA level by insecticide spray use (*p* < 0.01 both in total population and in females).

**Significance:**

Household insecticide spray was a predominant exposure source for pyrethroids at community level among Korean elders, warning more stringent control for frequently exposed environmental factors for pyrethroids including insecticide spray.

## Introduction

Pyrethroids are synthetic analogues of natural insecticidal compounds present in the extracts of pyrethrum flowers. Those have been reported to have high insecticidal activity and low mammalian toxicity compared with other classes of insecticides [1, 2]. For this reason, pyrethroid insecticides have been used to control insect pests in agricultural crops as well as in urban settings. Pyrethroids have been reported to increase adverse health consequences even at low-dose exposures, including obesity, respiratory, and developmental outcomes [2–5]. Based on the amount of pesticide used each year, in 2010 Korea was ranked second among 19 countries [6, 7]. Furthermore, pyrethroids were known to be among the most frequently used pesticide in Korea [7]. The general population are exposed to pyrethroids via direct and indirect ingestion, and dermal contact while common sources might include mosquito repellents, insecticidal sprays, and residues found in foods [8–10]. Because of this exposure nature, pyrethroids exposure in human is commonly measured internally in urine using their metabolites such as 3-phenoxybenzoic acid (3-PBA), 4-fluoro-3-phenoxybenzoic acid, cis-(2,2-dichlorovinyl)-2,2-dimethylcyclopropane-1-carboxylic acid, trans-(2,2-dichlorovinyl)-2,2-dimethylcyclopropane-1-carboxylic acid, and cis-(2,2-dibromovinyl)-2,2-dimethylcyclopropane-1-carboxylic acid [11, 12]. Because 3-PBA among the metabolites of pyrethroids was the most commonly detected, urinary 3-PBA levels have been used to determine the pyrethroids exposure [11, 12]. However, half-lives of pyrethroids-producing 3-PBA as its metabolite are short under 8–27 h depending on oral, respiratory, and dermal exposures, and thus the exposures need to be evaluated using repeated urinary measures data [13].

The population adults above age 60 are increasing in number worldwide, with an expected 22% of the world population to fall into this category by 2050 [14]. The Korea National Statistical Office has also predicted that Korea will be among countries termed “a super-aged society” by 2026 [15]. Elders are vulnerable to environmental chemicals, and exposure to environmental chemicals including pyrethroids can result in adverse health outcomes related to potentially shorter life expectancy in the elders [3, 16]. Moreover, pyrethroids exposure was reported to be associated with cognitive score linked with dementia in representative U.S. elders between 1999 and 2002 [17]. For these reasons, examining exposure to pyrethroids in the senior population might be important in Korea as well as other countries where elder populations are rapidly expanding. Nevertheless, the studies comparing pyrethroids exposure or 3-PBA levels in elders living in rural or urban areas are limited, with few studies reporting on potential routes of exposures, and viable sources of exposure [10, 12].

Therefore, we measured 3-PBA levels in urine samples collected from Korean elders and compared urinary 3-PBA concentrations between rural and urban elders. In addition, we assessed proportions over existing limits to determine pyrethroids exposure among Korean elders. We also evaluated potential sources of pyrethroid exposures, as surveyed using a questionnaire, relative to measure urinary 3-PBA levels.

## Methods

### Population

The Korean Elderly Environmental Panel study II (KEEP-II) was conducted as a subsequent study of KEEP-I to evaluate relations between exposure to environmental pollutions and health indicators of elders aged ≥60. For the KEEP-II, we recruited 1254 elders with 695 living in Asan (rural area) and 559 living in Seoul (urban area) from October 2012 to April 2015. In three surveys conducted at ~1-year intervals for KEEP-II (1st, October 2012 to February 2013; 2nd, January 2014 to April 2014; and 3rd, November 2014 to April 2015), we collected urine samples a maximum of three times per individual. Among 1254 subjects recruited, we excluded 15 from Asan who did not give urine samples, resulting in a final total of 1239 elders for the study analyses. Although there were an additional 15 subjects for whom we could not measure weight or height and thus could not determine their body mass index (BMI, kg/m^2^), we did not exclude the individuals from the final analyses because we could not find any association between BMI and 3-PBA levels. Six hundred and eighty elders living in the Asan area visited the elderly welfare center an average of 1.4 times during the study period: 495 (72.8%), 113 (16.6%), and 72 (10.6%) visiting one, two, and three times, respectively, providing a total of 937 urines. The 559 elders living in Seoul visited the elderly welfare center 2.1 times on average, with 165 (29.5%), 152 (27.2%), and 242 (43.3%) visiting one, two, and three times, respectively, providing a total of 1195 urine samples. Trained interviewers obtained demographic information including residence and lifestyle habits as well as potential pyrethroids exposure history from each participant using structured questionnaires. Our protocol was approved by the Seoul National University Hospital Institutional Review Board, South Korea (H-1209-006-424), and we conducted the study in accordance with guidelines set in the Declaration of Helsinki. Each participant provided written informed consent.

### 3-PBA level measurement

A total of 2132 urine samples were collected from 1239 elders. Those samples were stored on ice just after collection, transported to the laboratories, and then analyzed for 3-PBA level by a previously reported procedure [3]. Briefly, 3 mL of urine was sequentially extracted with 37% HCl, *n*-hexane, and 0.1-M NaOH solutions, and final extracted solution was dried in a nitrogen stream and re-dissolved with solvent [3]. Then, dissolved sample was used to detect 3-PBA level using gas chromatography mass spectrometer (Clarus 680T, Perkin Elmer, Waltham, MA, USA). The quality control and quality assurance were also conducted [3]. The limit of detection (LOD) for 3-PBA level differed depending on the survey with 0.014 ng/mL for the first survey, 0.013 ng/mL for the second, and 0.010 ng/mL for the third because we measured 3-PBA level in the samples just after we had completed the sampling for each survey. The number of samples with 3-PBA level under the LOD was 3 (0.4%) for the first survey, 7 (0.9%) for the second, and 2 (0.4%) for the third, and 3-PBA levels under the LOD were assigned as the default value of LOD divided by $\sqrt 2$, because our dataset for 3-PBA had relatively few data below the detection limit [18]. To control urinary dilution level, we measured urinary creatinine levels as well. For creatinine measurement, we used the kinetic Jaffe method (Cobas 8000 C702; Roche Diagnostics, Mannheim, Germany) by a commercially recommended procedure [3].

### Statistical analyses

The participant characteristics were examined at first visit, and the distribution of 3-PBA levels before and after correction for urinary creatinine was compared using Student’s *t* or analysis of variance (ANOVA) by population characteristics; sex (male or female), age (60–69, 70–79, 80–89, or 90–99 years), BMI (<18.5, 18.5–<23, 23–<25, or ≥25 kg/m^2^), smoking status (current smoker, ex-smoker, or nonsmoker), drinking status (current drinker, ex-drinker, or nondrinker), residence area (rural or urban), visit episode (the order of personal examination; first, second, or third), survey episode (survey duration; 1st, 2nd, or 3rd), surveyed season (spring, summer, fall, or winter), use frequency per week for insecticide spray (0–<3, 3–<7, or 7–20), and dietary intake such as consumption of vegetables (0–<1, 1–3, 4–6, or 7 days per week) and herbal refreshment (<1, 1–2, or 3 packs per day). Geometric mean (GM), standard error (SE), and selected percentile (10, 25, 50, 75, 90, or 95 percentile) values of 3-PBA were also calculated. Further, we assessed proportions over some limit values. Because there was no biological limit value for urinary 3-PBA, we assessed exceedance proportions based on previously reported reference guide I (1.7 ng/mL based on urinary excretion fraction to all pyrethroids), reference value derived from the 95th percentile of the representative populations (RV_95_) (2 ng/mL based on German Human Biomonitoring), RV_95_ obtained from representative Korean population (8.86 ng/mL based on Korean National Environmental Health Survey (KoNEHS)), and reference guide II (87 ng/mL based on weighted biomonitoring equivalent values relying on exposure guidance values for each pyrethroid as well as urinary excretion fraction to all pyrethroids) [3, 13, 19, 20].

Since participants had a maximum of three repeated visits with urine samples collected at each visit, individual mean 3-PBA levels were used in the analyses for the variability considering between- and within-individual variations of 3-PBA levels. Further, the variability trend of 3-PBA levels was compared between rural and urban areas. To evaluate how much portion between- or within-individual variation of 3-PBA levels could explain among total variation (between-individual variation plus within-individual variation), we calculated intra-class correlation (ICC) defined as the ratio of between-individual variation to total variation using a linear mixed effect model after log_2_-tranformation of 3-PBA level [3, 21]. The ICC value was also compared between rural and urban areas.

We evaluated pyrethroid exposure sources using questionnaire data. Surveyed questions included: “Did you use insecticide sprays at home?” and “If yes, how many times did you use those per week?”. The frequency per week was recorded as 0 for the subjects who answered “No” for the first question. To determine current occupational exposure, we asked the questions: “What is your job now?” and “Are you engaging in agriculture?”. The subjects who answered “Farmer” in first question or “Yes” in the second question were recoded as 1 for the variable “potential occupational exposure” and the other responses were recoded as 0. To identify the dietary intake regarding the consumption of vegetables and herbal refreshment, we asked the questions: “How often did you take vegetables per week?” and “How much did you take herbal refreshment (packs per day)?”. To evaluate pyrethroid exposure sources, 3-PBA level was creatinine-corrected to control urinary dilution, and the creatinine-corrected 3-PBA levels were log_2_-transformed for their normalization. And then relations between potential pyrethroid exposure sources examined using questionnaire data and urinary 3-PBA levels were evaluated using linear mixed effect models and generalized additive mixed models (GAMMs) for linear and nonlinear relationships, respectively. In both models, participants were treated as random effects, and several covariates, sex, age, smoking status, visit episode, and surveyed season, were adjusted because these covariates were associated with 3-PBA level in our study. For the sensitivity analyses, we adjusted for BMI, drinking status, and residence area (or plus potential occupational exposure (Yes or No)) as well as the previously mentioned covariates. In addition, we repeated the analyses after adjustment for urinary creatinine level (ng/mL) in the models, using log_2_ 3-PBA levels, which were not creatinine-corrected as an outcome variable, since urinary creatinine concentration could be different depending on the age, sex, race/ethnicity, BMI, and so on [22]. We also conducted the analyses after assigned 3-PBA levels under LODs as the default value of 0.014 ng/mL (maximum LOD value across the batches) divided by $\sqrt 2$ because of a potential error which could be produced by different LOD levels by surveys. Furthermore, we analyzed by sex, because of physiological differences and a potentially different working hours between males and females.

The R version 3.4.3 (The Comprehensive R Archive Network: http://cran.r-project.org) and SAS version 9.4 Enterprise (SAS Institute Inc., Cary, NC, USA) were used for all statistical analyses with significance criteria of two-sided *p* value < 0.05.

## Results

### Participant characteristics

Our participants included 377 males and 862 females (Table 1). At the first visit, mean age of our participants was 75.3 years ranged from 60 to 98 years. Most participants were ex-drinkers (79.3%) and nonsmokers (81.7%) (Table 1). The number of participants living in rural and urban areas was 680 (54.9%) and 559 (45.1%), respectively (Table 1). The distributions of smoking or drinking status and residence area were found to be different between males and females (*p* < 0.01 for all three variables). Participations of ex-smokers versus nonsmokers and current drinkers versus ex-drinkers in males were 40.8% versus 45.4% and 43.2% versus 56.8%, respectively, while most female participants were nonsmokers (97.6%) and ex-drinkers (89.2%) (Table 1). Moreover, males living in rural area more actively participated in the study compared with males living in urban area, with 69.0% and 31.0% for participation of males in Asan and Seoul, respectively, while participation of females was similar in Asan (48.7%) and Seoul (51.3%) (Table 1).

**Table 1:** Characteristics of participants.

Characteristic	Males	Females	Total
No. of participants (%)	377 (30.4)	862 (69.6)	1239 (100)
Mean age, year (min–max)	75.5 (60–98)	75.3 (60–92)	75.3 (60–98)
Height (cm), mean ± SE	165.1 ± 0.3	152.0 ± 0.2	156.0 ± 0.2
Weight (kg), mean ± SE	63.7 ± 0.4	55.4 ± 0.3	57.9 ± 0.3
BMI (kg/m^2^), no. (%)			
<18.5	14 (3.7)	36 (4.2)	50 (4.1)
18.5–< 23	148 (39.7)	311 (36.6)	459 (37.4)
23–< 25	119 (31.9)	200 (23.5)	319 (26.1)
≥25	92 (24.7)	304 (35.7)	396 (32.4)
Smoking status, no. (%)			
Current smoker	52 (13.8)	8 (0.9)	60 (4.8)
Ex-smoker	154 (40.8)	13 (1.5)	167 (13.5)
Nonsmoker	171 (45.4)	841 (97.6)	1012 (81.7)
Drinking status, no. (%)			
Current drinker	163 (43.2)	93 (10.8)	256 (20.7)
Ex-drinker	214 (56.8)	769 (89.2)	983 (79.3)
Nondrinker	0 (0)	0 (0)	0 (0)
Residence area, no. (%)			
Asan, rural	260 (69.0)	420 (48.7)	680 (54.9)
Seoul, urban	117 (31.0)	442 (51.3)	559 (45.1)
Visit number, no. (%)			
Once	245 (65.0)	415 (48.1)	660 (53.3)
Two times	67 (17.8)	198 (23.0)	265 (21.4)
Three times	65 (17.2)	249 (28.9)	314 (25.3)

*SE* standard error, *BMI* body mass index.

### 3-PBA levels by population characteristics and exceedance proportions for limit values of 3-PBA level

A total of 2120 (99.4%) urines among 2132 urine samples were detected over LOD, while 12 (0.6%) samples (3 in Asan at 1st visit, 6 in Asan and 1 in Seoul at 2nd visit, and 2 in Asan at 3rd visit) were detected below the LOD. Distributions of 3-PBA levels before and after correction for urinary creatinine by population characteristics were shown in Tables 2 and 3, respectively. The GM and SE, respectively, for 3-PBA levels in all urine samples were 1.19 and 0.03 ng/mL before correction for creatinine levels (Table 2) and 1.46 and 0.03 ng/mg-c after creatinine correction (Table 3). Because of potential differences in insecticide exposure between males and females, by age, between rural and urban areas, by survey episode (because of survey duration related to season), and by surveyed season, we compared urinary 3-PBA levels by these population characteristics as well as by basic characteristics including BMI, smoking status, and drinking status. Urinary 3-PBA levels showed significant differences by survey episode and surveyed season, with the highest exposure levels in the second survey and in spring (GM, 2.04 ng/mL at second survey and 1.60 ng/mL in spring, with *p* < 0.01 for both) (Table 2). This trend remained even after we corrected for creatinine levels (GM, 2.28 ng/mg-c at the second survey and 1.83 ng/mg-c in the spring, with *p* < 0.01 for both) (Table 3). Residence area showed a marginal significance before we corrected for creatinine levels (*p* = 0.08) (Table 2), but this trend disappeared after the correction (*p* = 0.12) (Table 3). On the other hands, females and nonsmokers showed significantly higher 3-PBA levels than those of males and current or ex-smokers, respectively, when the levels were corrected for creatinine levels (GM, 1.14 ng/mg-c in males and 1.60 ng/mg-c in females with *p* < 0.01; and 1.09 ng/mg-c in current smokers, 1.16 ng/mg-c in ex-smokers, and 1.54 ng/mg-c in nonsmokers with *p* = 0.02) (Table 3). Age also showed a marginal significance in trend test after correction for creatinine levels (*p* = 0.06), although it did not show any significance in general ANOVA (Table 3). However, BMI, drinking status, and residence area did not show any statistical relation with 3-PBA level (Tables 2 and 3). To explore general sources of pyrethroids exposure, we compared 3-PBA levels by frequency of insecticide spray use or dietary intake including consumption of vegetables and herbal refreshment. We found consistent difference of urinary 3-PBA levels by frequency of insecticide spray use in regardless of creatinine correction (*p* < 0.01 for both) (Tables 2 and 3). However, we did not find any other exposure sources for 3-PBA levels, including dietary intake (Tables 2 and 3).

**Table 2:** Urinary 3-PBA levels (ng/mL) by population characteristics.

				Selected percentiles						No. (%) ≥references^a^			
Subjects	Observation no.	GM ± SE	*p* value (*p*-for-trend)	10	25	50	75	90	95	Guide I	RV_95_^b^	RV_95_^c^	Guide II
Total population	2132	1.19 ± 0.03		0.28	0.57	1.24	2.59	4.98	7.59	834 (39.1)	702 (32.9)	79 (3.7)	1 (0.1)
Sex													
Male	574	1.09 ± 0.06	0.57	0.24	0.55	1.21	2.31	4.95	8.54	201 (35.0)	168 (29.3)	27 (4.7)	0 (0)
Female	1558	1.23 ± 0.04		0.29	0.58	1.25	2.70	5.04	7.42	633 (40.6)	534 (34.3)	52 (3.34)	1 (0.1)
Age (year)													
60–69	323	1.41 ± 0.10	0.12	0.31	0.73	1.41	3.27	6.08	8.84	137 (42.4)	121 (37.5)	16 (5.0)	0 (0)
70–79	1247	1.17 ± 0.04	(0.13)	0.28	0.55	1.24	2.55	5.04	7.32	491 (39.4)	405 (32.5)	42 (3.4)	1 (0.1)
80–89	534	1.14 ± 0.06		0.27	0.58	1.18	2.47	4.24	7.39	196 (36.7)	168 (31.5)	21 (3.9)	0 (0)
90–99	28	0.71 ± 0.18		0.11	0.25	0.79	2.19	3.80	4.56	10 (35.7)	8 (28.6)	0 (0)	0 (0)
BMI (kg/m^2^)													
<18.5	75	1.13 ± 0.16	0.41	0.23	0.54	1.51	2.32	5.07	8.94	32 (42.7)	23 (30.7)	3 (4)	0 (0)
18.5–<23	729	1.11 ± 0.05		0.26	0.54	1.19	2.49	5.03	7.42	280 (38.4)	241 (33.1)	20 (2.7)	0 (0)
23–<25	544	1.17 ± 0.06		0.27	0.54	1.22	2.53	4.69	7.49	220 (40.4)	179 (32.9)	23 (4.2)	0 (0)
≥25	769	1.29 ± 0.05		0.32	0.62	1.28	2.76	5.17	7.82	299 (38.9)	256 (33.3)	33 (4.3)	1 (0.1)
Smoking status													
Current smoker	88	0.89 ± 0.14	0.59	0.10	0.44	1.20	2.36	4.52	5.30	30 (34.1)	25 (28.4)	2 (2.3)	0 (0)
Ex-smoker	270	1.08 ± 0.08		0.23	0.57	1.18	2.15	4.49	7.53	89 (33.0)	72 (26.7)	11 (4.1)	0 (0)
Nonsmoker	1774	1.22 ± 0.03		0.29	0.58	1.25	2.67	5.08	7.89	715 (40.3)	605 (34.1)	66 (3.7)	1 (0.1)
Drinking status													
Current drinker	469	1.13 ± 0.06	0.29	0.24	0.54	1.24	2.49	5.07	7.90	172 (36.7)	148 (31.6)	16 (3.4)	0 (0)
Ex-drinker	1663	1.21 ± 0.03		0.29	0.59	1.24	2.59	4.98	7.55	662 (39.8)	554 (33.3)	63 (3.8)	1 (0.1)
Residence area													
Asan, rural	937	1.11 ± 0.04	0.08	0.27	0.57	1.19	2.44	4.69	7.42	354 (37.8)	286 (30.5)	31 (3.3)	0 (0)
Seoul, urban	1195	1.25 ± 0.04		0.28	0.56	1.30	2.76	5.22	7.97	480 (40.2)	416 (34.8)	48 (4.0)	1 (0.1)
Survey episode													
1st (2012.10.29 to 2013.02.28)	799	0.98 ± 0.04	<0.01	0.27	0.54	1.02	1.89	3.36	5.04	234 (29.3)	178 (22.3)	17 (2.1)	0 (0)
2nd (2014.01.13 to 2014.04.30)	783	2.04 ± 0.08		0.57	1.09	2.24	4.17	7.49	9.84	478 (61.1)	420 (53.6)	51 (6.5)	1 (0.1)
3rd (2014.11.18 to 2015.04.03)	550	0.73 ± 0.04		0.18	0.35	0.76	1.53	3.13	4.25	122 (22.2)	104 (18.9)	11 ((2.0)	0 (0)
Surveyed season													
Spring (March to May)	451	1.60 ± 0.09	<0.01	0.35	0.77	1.81	3.50	5.66	7.90	234 (51.9)	206 (45.7)	17 (3.8)	1 (0.2)
Summer (June to August)	0	–		–	–	–	–	–	–	–	–	–	–
Fall (September to November)	161	0.74 ± 0.06		0.20	0.35	0.76	1.40	2.38	2.77	29 (18.0)	22 (13.7)	4 (2.5)	0 (0)
Winter (December to February)	1520	1.14 ± 0.04		0.27	0.56	1.18	2.47	4.89	7.93	571 (37.6)	474 (31.2)	58 (3.8)	0 (0)
Insecticide spray use (frequency per week)													
0–<3	1613	1.11 ± 0.03	<0.01	0.27	0.54	1.16	2.42	4.31	6.65	591 (36.6)	487 (30.2)	47 (2.9)	0 (0)
3–<7	247	1.48 ± 0.11		0.38	0.69	1.64	3.32	5.63	8.15	117 (47.4)	105 (42.5)	9 (3.6)	0 (0)
7–20	272	1.45 ± 0.12		0.29	0.67	1.46	3.55	8.25	11.12	126 (46.3)	110 (40.4)	23 (8.5)	1 (0.4)
Vegetable consumption (days per week)													
0–<1	102	1.16 ± 0.14	0.68	0.33	0.66	1.32	2.53	3.99	5.21	44 (43.1)	35 (34.3)	3 (2.9)	0 (0)
1–3	224	1.19 ± 0.09		0.23	0.59	1.34	2.54	4.83	6.80	93 (41.5)	72 (32.1)	7 (3.1)	0 (0)
4–6	178	1.12 ± 0.11		0.25	0.61	1.33	2.47	5.03	6.65	72 (40.4)	59 (33.1)	5 (2.8)	0 (0)
Everyday	1600	1.20 ± 0.04		0.28	0.56	1.22	2.67	5.18	7.94	620 (38.8)	532 (33.3)	63 (3.9)	1 (0.1)
Herbal refreshment consumption (packs per day)													
<1	68	1.61 ± 0.20	0.73	0.38	0.86	1.70	2.67	6.61	8.83	35 (51.5)	28 (41.2)	3 (4.4)	0 (0)
1–2	109	1.14 ± 0.13		0.24	0.56	1.04	2.94	5.35	5.85	42 (38.5)	37 (33.9)	2 (1.8)	0 (0)
3	1931	1.18 ± 0.03		0.28	0.57	1.24	2.58	4.95	7.82	753 (39.0)	633 (32.8)	73 (3.8)	1 (0.1)

*p* values were obtained using *t*-test or ANOVA. *p* value for trend was obtained using linear regression. The limit of detection (LOD) level for 3-PBA at first, second, and third surveys was 0.014, 0.013, and 0.010 ng/mL, respectively.

*3-PBA* 3-phenoxybenzoic acid, *GM* geometric mean, *SE* standard error.

^a^Exceedance proportion for the previously reported limit values including reference guide I (1.7 ng/mL).

^b^Reference value based on 95th percentile (RV95, 2 ng/mL based on German Human Biomonitoring).

^c^Reference value based on 95th percentile (RV95, 8.86 ng/mL based on Korean National Environmental Health Survey), and reference guide II (87 ng/mL) [3, 13, 19, 20].

**Table 3:** Urinary 3-PBA levels (ng/mg-c) by population characteristics.

				Selected percentiles					
Subjects	Observation no.	GM ± SE	*p* value (*p*-for-trend)	10	25	50	75	90	95
Total population	2132	1.46 ± 0.03		0.42	0.80	1.47	2.82	5.19	7.54
Sex									
Male	574	1.14 ± 0.05	<0.01	0.32	0.66	1.17	2.20	3.97	6.46
Female	1558	1.60 ± 0.04		0.46	0.88	1.62	3.04	5.50	7.68
Age (year)									
60–69	323	1.69 ± 0.10	0.15	0.45	0.92	1.70	3.33	6.10	9.07
70–79	1247	1.46 ± 0.42	(0.06)	0.42	0.81	1.48	2.81	5.05	7.24
80–89	534	1.38 ± 0.06		0.42	0.72	1.41	2.66	4.91	7.56
90–99	28	0.85 ± 0.17		0.20	0.33	0.88	1.96	3.32	4.38
BMI (kg/m^2^)									
<18.5	75	1.57 ± 0.19	0.87	0.33	0.98	1.67	3.14	6.03	7.56
18.5–<23	729	1.41 ± 0.56		0.39	0.78	1.43	2.70	5.04	7.25
23–<25	544	1.45 ± 0.07		0.42	0.74	1.50	2.81	4.98	7.70
≥25	769	1.52 ± 0.06		0.44	0.84	1.49	2.94	5.45	7.41
Smoking status									
Current smoker	88	1.09 ± 0.16	0.02	0.23	0.71	1.33	2.51	3.97	5.99
Ex-smoker	270	1.16 ± 0.08		0.33	0.61	1.17	2.13	3.86	6.03
Nonsmoker	1774	1.54 ± 0.04		0.44	0.83	1.53	2.93	5.35	7.60
Drinking status									
Current drinker	469	1.33 ± 0.07	0.51	0.36	0.68	1.33	2.73	5.53	7.70
Ex-drinker	1663	1.50 ± 0.04		0.43	0.83	1.52	2.85	5.10	7.50
Residence area									
Asan, rural	937	1.42 ± 0.05	0.12	0.39	0.82	1.48	2.78	4.84	7.13
Seoul, urban	1195	1.49 ± 0.04		0.42	0.77	1.47	2.89	5.53	7.93
Survey episode									
1st (2012.10.29 to 2013.02.28)	799	1.32 ± 0.04	<0.01	0.44	0.75	1.31	2.25	3.83	6.02
2nd (2014.01.13 to 2014.04.30)	783	2.28 ± 0.08		0.85	1.37	2.32	3.97	6.64	8.81
3rd (2014.11.18 to 2015.04.03)	550	0.90 ± 0.04		0.26	0.46	0.87	1.74	3.49	6.73
Surveyed season									
Spring (March to May)	451	1.83 ± 0.09	<0.01	0.51	0.97	1.89	3.61	6.05	7.93
Summer (June to August)	0	–		–	–	–	–	–	–
Fall (September to November)	161	1.01 ± 0.07		0.34	0.56	0.98	1.61	2.82	4.47
Winter (December to February)	1520	1.42 ± 0.04		0.41	0.77	1.45	2.70	4.97	7.43
Insecticide spray use (frequency per week)									
0–<3	1613	1.39 ± 0.04	<0.01	0.41	0.76	1.42	2.64	4.65	6.76
3–<7	247	1.71 ± 0.11		0.52	1.01	1.82	3.19	5.92	7.43
7–20	272	1.72 ± 0.13		0.45	0.85	1.72	3.65	7.56	11.25
Vegetable consumption (days per week)									
0–<1	102	1.49 ± 0.16	0.67	0.45	0.90	1.62	2.72	3.53	4.47
1–3	224	1.48 ± 0.10		0.42	0.86	1.63	2.77	4.47	6.61
4–6	178	1.36 ± 0.12		0.42	0.90	1.49	2.37	5.03	6.74
Everyday	1600	1.48 ± 0.04		0.42	0.77	1.47	2.90	5.43	7.82
Herbal refreshment consumption (packs per day)									
<1	68	1.89 ± 0.21	0.76	0.63	1.25	1.94	3.18	5.71	11.24
1–2	109	1.46 ± 0.14		0.50	0.83	1.36	2.80	5.21	6.48
3	1931	1.46 ± 0.04		0.42	0.79	1.47	2.84	5.24	7.56

*p* values were obtained using *t*-test or ANOVA. *p* value for trend was obtained using linear regression. The limit of detection (LOD) level for 3-PBA at first, second, and third surveys was 0.014, 0.013, and 0.010 ng/mL, respectively.

*3-PBA* 3-phenoxybenzoic acid, *GM* geometric mean, *SE* standard error.

We derived exceedance proportions for existing limits including the reference guides or reference values derived on RV_95_. Because the existing limit values were not available after correction for creatinine levels, we derived exceedance proportions for reference guides I and II, and reference value (RV_95_) in German or Korean population before correction for creatinine levels as shown in Table 2. Among 2132 urinary samples, only one showed 3-PBA level over reference guide II (87 ng/mL). However, a total of 79 (3.7%), 702 (32.9%), and 834 (39.1%) samples had 3-PBA levels over Korean RV_95_ (8.86 ng/mL), German RV_95_ (2 ng/mL), and reference guide I (1.7 ng/mL), respectively (Table 2).

### Proportion of elders exposed to high level of pyrethroids and ICC values

**Fig. 1:**
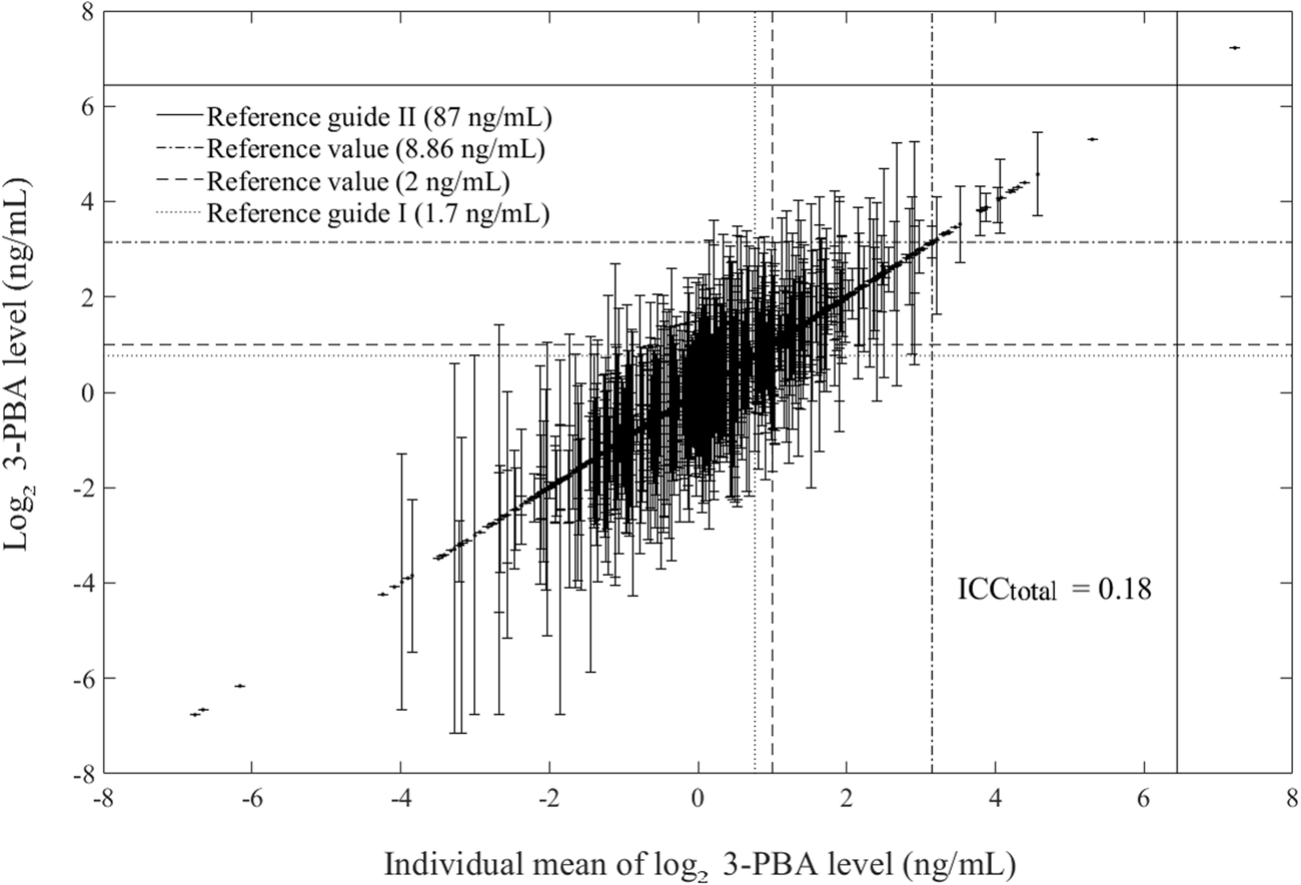
Variation by individual mean 3-PBA level. Both axes were expressed in log_2_ scale of 3-PBA (ng/mL). Vertical lines represented individual ranges—minimum and maximum of individual 3-PBA levels. Intra-class correlation (ICC) was calculated using a linear mixed effect model.

To determine whether Korean elders have been exposed to high level of pyrethroids compared with general population, we derived exceedance proportions for the above limit values using individual means of repeated 3-PBA levels. The numbers of elders with mean 3-PBA levels over the limits were 534 (43.1%) for reference guide I (1.7 ng/mL), 446 (36.0%) for German RV_95_ (2 ng/mL), 27 (2.2%) for Korean RV_95_ (8.86 ng/mL), and 1 (0.001%) for reference guide II (87 ng/mL) in the total population. By residence area, the numbers were 271 (39.9%), 223 (32.8%), 18 (2.6%), and 0 (0%), respectively, in Asan and 205 (36.7%), 169 (30.2%), 9 (1.6%), and 1 (0.2%), respectively, in Seoul (Fig. 1).

In the present study, we determined the ICC values after log_2_-transformation because of considerable differences in within-individual variations of 3-PBA level. With repeated log_2_-transformed 3-PBA levels, the ICC value for the total population was 0.18, with 0.20 in Asan and 0.15 in Seoul (Fig. 1). When we used log_2_-transformed creatinine-corrected 3-PBA levels instead, the ICC value was 0.14 for total population, 0.17 for the elders living in Asan, and 0.12 for those living in Seoul.

### Comparison of 3-PBA levels with previous studies

No significant difference in 3-PBA levels between rural and urban elders was found, although the level was slightly higher among the elders living in Seoul. Consequently, we compared 3-PBA levels for rural and urban Korean elders with the levels in adults including elders in other countries (Table 4) [9, 19, 23–45]. Except for the study conducted in Italy with a relatively high LOD for 3-PBA level and considering for only urines with 3-PBA levels over the LOD [31], Korean elders had the highest 3-PBA levels of all countries regardless of correction for creatinine levels (GM 1.19–1.47 ng/mL in Korean adults versus 0.20–0.97 ng/mL in other countries), although the KoNEHS showed slightly higher 3-PBA levels than those in our study (Table 4) [19].

**Table 4:** Previous studies for urinary 3-PBA levels in general adult population and comparison with the present study.

Country (study year)	Targets	Age	Subject number	Sample number	%^a^	Geometric mean (median)
Canada (2007–2009) [23]	National study for Canadian Health Measures Survey (CHMS)	20–79	3439	3439	99.4	0.317 ng/mg-c (0.272 ng/mg-c)
Caribbean (2008–2011) [24]	Pregnant women	27^b^	297	295	100	0.54 ng/mL (–)
China (2013–2015) [25]	Shanghai resident women	≥20	615	615	99.0	0.51 ng/mL and 0.73 ng/mg-c
China (2011–2013) [26]	Pregnant women	≥18	374	374	90.4	0.46 ng/mL and 1.09 ng/mg-c (0.48 ng/mL and 1.14 ng/mg-c)
China (2009–2010) [9]	Pregnant women in agricultural area	17–45	1149	1149	>98.3	0.97 ng/mL and 1.53 ng/mg-c (1.01 ng/mL, 1.55 ng/mg-c)
France (2011–2013) [27]	General population consumed organic or conventional food	≥18	150/150	150/150	23/35	0.0201/0.0282 ng/mg-c
France (2011) [28]	Pregnant women	≥18	1077	1077	100	0.36 ng/mL and 0.50 ng/mg-c (0.36 ng/mL and 0.50 ng/mg-c)
France (2002–2006) [29]	Pregnant women	–	287	205	30.2	<LOD, 0.008
Ghana (2014) [30]	Pregnant women in rural area		17	49	75.5	0.23 ng/mL
Italy (1993–1998) [31]	Healthy adults in Florence and Ragusa	35–64	69	69	53.6	1.58 ng/mL^d^ (–)
Japan (2009–2011) [32]	Pregnant women	20–50	222	231	–	0.334 ng/mL, 0.376 ng/mL-SG, and 0.363 ng/mg-c (0.351 ng/mL, 0.361 ng/mL-SG, and 0.338 ng/mg-c)
Japan (2005) [33]	Pregnant women	39–85	448	448	98	0.29 ng/mL and 0.40 ng/mg-c (0.29 ng/mL and 0.36 ng/mg-c)
Japan (2005) [34]	Rural/suburban males	63.9 ± 0.83/49.3 ± 1.5^c^	143/66	143/66	–/–	0.32 ng/mg-c (0.28 ng/mg-c)/0.49 ng/mg-c (0.43 ng/mg-c)
Poland (2010–2011) [35]	Rural/urban residents	18–77	56/134	56/134	80	0.317/0.203 ng/mL and 0.189/0.121 ng/mg-c (–)
Puerto rico (2010–2012) [36]	Pregnant women	18–40	54	141	46	0.20 ng/mL (<0.1 ng/mL)
South Korea (2009–2011) [19]	Korean National Environmental Health Survey (KoNHES)	≥19	6232	6232	99.8	1.47 ng/mL (1.55 ng/mL)
South Korea (2008–2010) [37]	Pregnant women	–	578	578	98.96	0.976 ng/mg-c (0.956 ng/mg-c)
South Africa (2012–2013) [38]	Venda Health Examination of Mothers, Babies and the Environment (VHEMBE)	≥18	705	694	100	0.712 ng/mL-SG (0.700 ng/mL-SG)
Thailand (2017) [39]	Nonfarm workers/farmers living in agricultural community	18–65	100/300	100/300	36.8/28.8	16.7/20.2 ng/mL (–)
USA (2016–2017) [40]	Urban/suburban pregnant women	18–35	20	20	70	0.55 ng/mL
USA (2009–2011) [41]	Repeatedly collected urines for longitudinal study in North Carolina	19–50	50	2472	74	0.96 ng/mL and 1.07 ng/mL-SG (0.88 ng/mL and 0.96 ng/mL-SG)
USA (2007–2010) [42]	National Health and Nutritional Examination Survey (NHANES)	20–79	2796	2796	72.0	0.41 ng/mL and 0.44 ng/mg-c (0.40 ng/mL and 0.40 ng/mg-c)
USA (2004) [43]	New York City Health and Nutrition Examination Survey (NYC HANES)	≥20	2000	1452	58.5	0.76 ng/mL and 0.75 ng/mg-c
USA (2000–2003) [44]	Males who visited fertility center	20–54	159	159	79	0.62 ng/mL and 0.47 ng/mg-c
USA (1999–2000, 2001–2002) [45]	National Health and Nutritional Examination Survey (NHANES)	20–59 (1999–2000)/20–59 (2001–2002)/≥60 (2001–2002)	833/1128/509	833/1128/509	64.2/75.8/70.3	0.267 ng/mL and 0.246 ng/mg-c (0.23 ng/mL and 0.26 ng/mg-c)/0.314 ng/mL and 0.311 ng/mg-c (0.27 ng/mL and 0.30 ng/mg-c)/0.303 ng/mL (0.32 ng/mL)
Present study (2012–2015)	Korean Elderly Environmental Panel study (KEEP)	60–98	1239	2132	99.4	1.19 ng/mL and 1.46 ng/mg-c (1.24 ng/mL and 1.47 ng/mg-c)

*SG* specific gravity.

^a^Percentage of urine samples with detectable levels of 3-PBA.

^b^Mean.

^c^Mean ± standard deviation (SD).

^d^Mean calculated from only 37 urines detected over the LOD (0.53555 ng/mL calculated from 2.5 nmol/L).

### Relationships between potential pyrethroid exposure sources and 3-PBA level

To assess whether household insecticide spray use is a predominant pyrethroids exposure source among general elder individuals in Korea, we estimated the relationships between frequency of potential pyrethroids exposures and log_2_-transformed creatinine-corrected 3-PBA level. As shown in Table 5, the frequency of household insecticide spray use was significantly associated with urinary 3-PBA level (*β* = 0.03 and *p* = 0.02) and the association was apparent in females rather than male participants (*β* = 0.03 and *p* = 0.36 for males; and *β* = 0.03 and *p* = 0.03 for females) (Table 5). This trend remained after additional adjustment for BMI, drinking status, and residence area (or plus potential occupational exposure) (Appendix Tables A1 and A2), after adjustment for urinary creatinine level (ng/mL) in the models (Appendix Table A3), or after assignment of 3-PBA levels under LODs as the default value of maximum LOD value divided by $\sqrt 2$ (Appendix Table A4). However, we did not find any other pyrethroids exposure sources except for household insecticide spray, even though we explored dietary intake including consumption of vegetables and herbal refreshment (Appendix Table A5).

**Table 5:** Relations of insecticide spray use (frequency per week) with log_2_-transformed urinary 3-PBA levels (ng/mg-c).

Target	Observation no.	*β*	SE	Lower 95% CI	Upper 95% CI	*p* value
Total	2132	0.03	0.01	0.004	0.06	0.02
Males	574	0.03	0.03	−0.03	0.09	0.36
Females	1558	0.03	0.01	0.003	0.06	0.03

Relation of insecticide spray use with log_2_-transformed 3-phenoxybenzoic acid (3-PBA) level was estimated using linear mixed effect model after adjustment for sex, age, smoking status, survey episode, and surveyed season.

*3-PBA* 3-phenoxybenzoic acid, *SE* standard error, *CI* confidence interval.

**Fig. 2:**
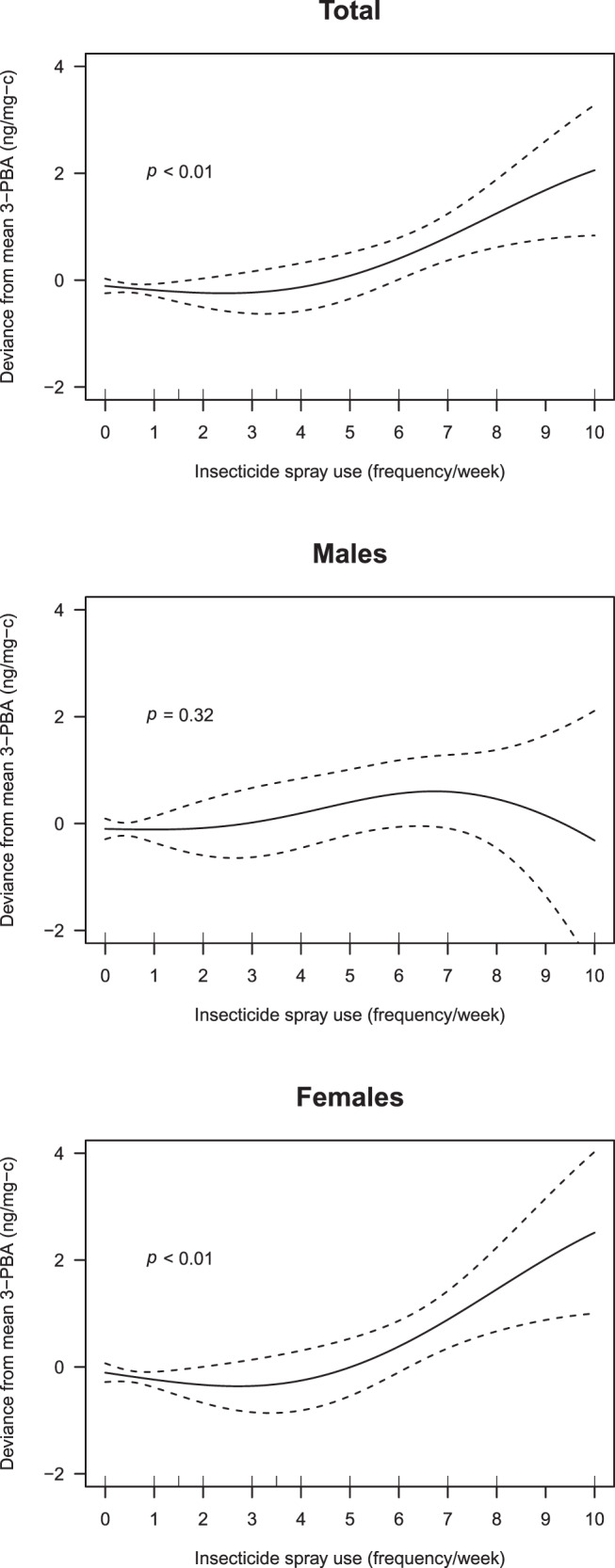
A penalized regression spline of insecticide spray use (frequency/week) on urinary 3-PBA level (ng/mg-c) after adjustment for sex, age, smoking status, survey episode, and surveyed season. *p* values were obtained using generalized additive mixed models (GAMMs).

To evaluate the nonlinear relationships between potential pyrethroid exposure sources and urinary 3-PBA level, we conducted penalized regression splines using the GAMM. We found a strong nonlinear relationship for total elder population, showing a weak J-shape change of 3-PBA level by insecticide spray use and no change in level by increase of insecticide spray use at the lower end of exposure; over a potential threshold, we found a strong increase in 3-PBA level by increase of insecticide spray use with statistical significance (*p* < 0.01) (Fig. 2). This relationship was more apparent in female participants (*p* < 0.01) (Fig. 2), and we did not find it in males except for a weak relationship between insecticide spray use and 3-PBA level at the lower end of exposure (*p* = 0.32) (Fig. 2). This trend held even after additional adjustment for BMI, drinking status, and residence area (or plus potential occupational exposure) (Appendix Figs. A1 and A2), after adjustment for urinary creatinine level (ng/mL) in the models (Appendix Fig. A3), or after assignment of 3-PBA levels under LODs as the default value of maximum LOD value divided by $\sqrt 2$ (Appendix Fig. A4).

## Discussions

In the present study, we measured urinary 3-PBA levels in Korean elders living in rural and urban areas and investigated relations between potential pyrethroid exposure sources and urinary 3-PBA level. We also derived exceedance proportions for existing limit values and evaluated between- and within-individual variations in 3-PBA levels.

In a previously published study, we found a significant association of urinary 3-PBA level with pulmonary function reduction in Korean urban elders [3]. Because we found that Korean urban elders were exposed to high level of pyrethroids in the previous study [3], as a consecutive action we compared urinary 3-PBA levels in rural elders with those in urban elders, assessed proportions over existing limits, and then evaluated potential sources of pyrethroid exposures in the present study.

Because humans could be exposed to 3-PBA itself produced from natural degradation of environmental pyrethroids, the urinary 3-PBA levels do not represent only the pyrethroid exposures but also the exposures to a degradation product itself. Nevertheless, urinary 3-PBA level is a target currently used to explore level of pyrethroids exposed to humans. Moreover, urinary 3-PBA levels are noteworthy because 3-PBA itself was reported to exhibit immunotoxicity and increase cellular reactive oxygen species level [46, 47].

In the present study, we compared our 3-PBA levels with the levels in previously reported studies. The study conducted in Italy [31] reported 3-PBA levels based on the unit nmol/day using 24-h urine as main results. Although the study showed 1.58 ng/mg-c of 3-PBA level, this study targeted only 37 urine samples with 3-PBA levels over a LOD of 3-PBA (2.5 nmol/L) among 69 urines (53.6%). However, the LOD was 0.53555 ng/mL based on molecular weight 214.22 g/mol and it was relatively very high compared with those in other studies including ours (≤0.014 ng/mL). The low proportion of urines with 3-PBA levels over the LOD and calculation of average 3-PBA level considering for only 3-PBA levels over the LOD could lead to overestimation of pyrethroids exposure in general population, indicating no comparability with data obtained from other previous studies as well as the present study.

Although RV_95_ is a statistical value and thus it does not have any meaning for biological health effect, reference guides I and II used in the present study could be considered as screening values for the assessment of biomonitoring data corresponding to current exposure guidance values such as reference doses or concentrations (RfDs or RfCs) or tolerable daily intakes (TDIs) [13]. Based on our high exceedance proportions for reference guide I and German RV_95_ with high levels of 3-PBA in our elder population and evidence for high 3-PBA levels in general Korean adults on the KoNEHS, Korean elders were expected to be exposed to high level of pyrethroids. Therefore, we first explored which characteristics affected urinary 3-PBA level. We found associations of 3-PBA levels with sex, age, smoking status, survey episode, and surveyed season but not with BMI, drinking status, or residence area. Younger elders had higher exposure than that among their elder counterparts, and this was a consistent result by working age. However, females showed significantly higher 3-PBA level than did male participants, contrary to our expectation that males might work longer hours and thus face more exposure to pyrethroids. This finding might be plausible considering that the men were elders and might have already retired. Further, it may be slow indoor degradation with respect to the outdoor environment, which was due to reduced influence of solar radiation, low humidity, poor house cleaning, etc. [48], considering for much time spent at the home in females compared with males. Survey episode and surveyed season were associated with each other because we conducted each survey during short periods within specific seasons (each survey included in a survey episode and surveyed season overlapped at most points based on survey time). Thus, both survey episode and surveyed season might have been related to insecticide use by season showing an association with 3-PBA levels. In contrast, we found no association between residence area and 3-PBA levels. Contrary to our expectation that elders living in a rural area would be more exposed to pyrethroids than those in the urban area due to a greater prevalence of occupational pesticide use, findings suggest that persons living in cities face potentially higher pyrethroids exposure [10, 35]. From an additional survey of 400 rural elders, we found a current agricultural history for 148 (37%) rural elders among 400 [49], but 3-PBA level was higher in urban elders without occupational exposure history than in the rural elders of the present study, indicating potentially higher environmental rather than occupational pyrethroids exposure. We could have expected this given that our participants were elders without hard working time even though they could work. Moreover, pyrethroids have been known to enter the human body environmentally in different ways including through consumption of nuts, beans, and vegetables, in textile and residential applications, and household use of mosquito repellents [8, 10]. In fact, high levels of pyrethroids exposure were reported to be largely due to the widespread use of household pesticides such as mosquito repellents [9]. Therefore, we attempted to explore nonoccupational exposure sources for pyrethroids and found a positive association between urinary 3-PBA level and household use of insecticide spray such as mosquito repellents, again indicating that the elder population in the present study had higher nonoccupational rather than occupational pyrethroids exposure. However, we did not find any other environmental pyrethroids exposure sources except for mosquito repellents, even though we explored dietary intake including consumption of vegetables and herbal refreshment as well as variables such as drinking status already mentioned. Furthermore, in the present study, the size for the effect of household insecticide spray use on 3-PBA levels looks to be small based on the *β* values. However, considering log_2_-transformation of 3-PBA level, creatinine-corrected 3-PBA level increased by 102% at every increment of insecticide spray use frequency, indicating that household insecticide spray use in Korean elders is a predominant pyrethroids exposure factor.

Because 3-PBA levels were higher in general in female participants than in male participants of our study, we further evaluated the relationship between insecticide spray use and 3-PBA level by sex. We found a significant positive association of insecticide spray use with 3-PBA level in female elders only, with the same positive direction at the lower end of insecticide use in males, although the association was not significant. This association remained after several sensitivity analyses, and the strong J-shape change in 3-PBA level by increase of pyrethroids exposure frequency we found in our female participants was consistent with prevalent relationships between potentially harmful exposures and adverse outcomes including bioindicators. However, this pattern should be further studied because the slope decreased with insecticide use more than ten times per week; it should be noted that the confidence interval in this range of insecticide use was too broad because of the small number of subjects in this range. In addition, several epidemiologic studies have shown associations between urinary 3-PBA levels and adverse symptoms such as adiposity in 4-year-old children and lung function decline in the elders aged 60 or over only in females and not in males [3, 4]. These sex-dependent effects on health outcomes could be because 3-PBA has shown estrogenic and antiestrogenic activities in various assays [50–52]. The endocrine disruption potential of 3-PBA was also found in several cross-sectional studies for representative Koreans and for normal males who visited hospital for vasectomy [53, 54]. Urinary 3-PBA level was associated with thyroid hormones in Korean adults [53] and with sex hormones such as follicle stimulating hormone, testosterone, and free androgen in normal adult males [54]. However, the endocrine disruption potential of 3-PBA should be further confirmed because the study results concluded using in vitro systems or cross-sectionally could not guarantee endocrine disruption potential at environmentally relevant exposure in vivo. Furthermore, the difference in 3-PBA levels between male and female elders and the association of insecticide spray use with 3-PBA level that we found only in females of our study should be further examined because our results were related to exposure, not with health outcomes.

We calculated ICCs for the total population, the elders in Asan, and the elders in Seoul, and found slightly higher values in Asan than in Seoul; this trend remained even after we used creatinine-corrected 3-PBA levels, although all ICC values were generally smaller than those before correction for urinary creatinine level. This finding means that within-individual variations in 3-PBA in Seoul were larger than the variations in Asan, making the between-individual variations in 3-PBA level in Seoul smaller than those in Asan. Furthermore, we calculated ICCs after log_2_-transformation because of bigger within-individual variations of 3-PBA level in individuals with higher 3-PBA level than in those with lower level. After log_2_-transformation of 3-PBA levels, we obtained smaller and more stable ICCs than those before the log_2_-transformation in both rural and urban areas (ICC_Total-before_ = 0.61 vs. ICC_Total-after_ = 0.18; ICC_Asan-before_ = 0.37 vs. ICC_Asan-after_ = 0.20; and ICC_Seoul-before_ = 0.76 vs. ICC_Seoul-after_ = 0.15). In addition, the elders with a relatively high level of 3-PBA showed a consistent exposure (smaller within-individual variation in 3-PBA) as shown in Fig. 1.

In relation to survey episode, GM of 3-PBA level at 2nd round was significantly higher than those at other rounds regardless of creatinine correction. In our study, among 660 participants who visited only once, 276 (42%) participants visited at 2nd round survey. The 3-PBA level in elders who participated only in 2nd round survey was significantly higher compared with those at the other round surveys (GM of 3-PBA levels, 0.89 ng/mL at 1st round, 1.96 ng/mL at 2nd round, and 0.77 ng/mL at 3rd round, *p* < 0.01; and 1.24 ng/mg-c at 1st round, 2.17 ng/mg-c at 2nd round, and 0.86 ng/mg-c at 3rd round, *p* < 0.01). A relatively high GM of 3-PBA levels at 2nd round survey of our study could be due to a high participation rate of elders with high level of 3-PBA exposure at 2nd round survey.

Urinary creatinine could be no longer linearly related to the diuresis at extreme concentrations of urinary creatinine <0.3 g/L or >3 g/L [55]. In the present study, the number of urines with creatinine level <0.3 g/L or >3.0 g/L was 120 and 17, respectively. However, the trend of our results was not changed even after urines with extreme creatinine levels were excluded (data not shown here).

Our study had several strengths. We used repeated urinary 3-PBA measures to reflect pyrethroids exposure. This longitudinal panel design could present the temporal variations in pyrethroids exposure, which can easily change over time. Moreover, with this study design we could investigate each subject as his or her own control and evaluate short-term effects of pyrethroids exposure using 3-PBA as a covariate changing temporally in individuals. Furthermore, we are the first to identify an environmental (nonoccupational) source for pyrethroids exposure among Korea’s elders. However, our study had limitations as well. In the present study, we collected the information for insecticide spray use from questionnaire and thus it was impossible to identify the time interval between insecticide spray use and urine collection. Although behavioral pattern for insecticide spray use could not be easily changed, the time interval between the insecticide spray use and urine collection may greatly affect the urinary levels of 3-PBA due to the rapid metabolism of pyrethroids in humans. Furthermore, our participants were not representative because we targeted only one rural and one urban areas, although our 3-PBA levels were comparable with those measured in adults including the elders in the KoNEHS. Therefore, other environmental sources related to pyrethroids exposure should be further investigated in representative elder population.

## Conclusion

In summary, the Korean elders were exposed to high level of pyrethroids, and insecticide spray use was a predominant environmental exposure source. Therefore, further investigations are needed on pyrethroid exposure sources in the Korean elders and more stringent control for frequently exposed environmental factors including insecticide spray use is necessary to protect the elders, who are vulnerable to environmental chemicals including pyrethroids.

## Supplementary information

Appendix A
