# Supplementary material for: Household insecticide use and urinary 3-phenoxybenzoic acid levels in an elder population: a repeated measures data
Source: J Expo Sci Environ Epidemiol. 2020 Oct 6;31(6):1017–31. doi: 10.1038/s41370-020-00276-3 (PMC8589668; doi:10.1038/s41370-020-00276-3)

Appendix A

Household insecticide use and urinary 3-phenoxybenzoic acid levels in an elder population: a repeated measures data

Appendix Tables ***A2***

Table A1 Relations of insecticide spray use (frequency per week) with log_2-transformed urinary 3-PBA levels (ng/mg-c) after adjustment for sex, age, smoking status, survey episode, surveyed season, BMI, drinking status, and residence area ***A2***

Table A2 Relations of insecticide spray use (frequency per week) with log_2-transformed urinary 3-PBA levels (ng/mg-c) after adjustment for sex, age, smoking status, survey episode, surveyed season, BMI, drinking status, residence area, and potential occupational exposure ***A3***

Table A3 Relations of insecticide spray use (frequency per week) with log_2-transformed urinary 3-PBA levels (ng/ml) after adjustment for sex, age, smoking status, survey episode, surveyed season, and urinary creatinine level ***A4***

Table A4 Relations of insecticide spray use (frequency per week) with log_2-transformed LOD-corrected 3-PBA levels (ng/mg-c) after adjustment for sex, age, smoking status, survey episode, and surveyed season ***A5***

Table A5 Relations of the consumption of vegetables and herbal refreshment with log_2-transformed urinary 3-PBA levels (ng/mg-c) after adjustment for sex, age, smoking status, survey episode, and surveyed season ***A6***

Appendix Figures ***A7***

Figure A1 A penalized regression spline of insecticide spray use (frequency/week) on urinary 3-PBA level (ng/mg-c) after adjustment for sex, age, smoking status, survey episode, surveyed season, BMI, drinking status, and residence area ***A7***

Figure A2 A penalized regression spline of insecticide spray use (frequency/week) on urinary 3-PBA level (ng/mg-c) after adjustment for sex, age, smoking status, survey episode, surveyed season, BMI, drinking status, residence area, and potential occupational exposure ***A8***

Figure A3 A penalized regression spline of insecticide spray use (frequency/week) on urinary 3-PBA level (ng/ml) after adjustment for sex, age, smoking status, survey episode, surveyed season, and urinary creatinine level ***A9***

Figure A4 A penalized regression spline of insecticide spray use (frequency/week) on LOD-corrected 3-PBA levels (ng/mg-c) after adjustment for sex, age, smoking status, survey episode, and surveyed season ***A10***

Table A1 Relations of insecticide spray use (frequency per week) with log_2-transformed urinary 3-PBA levels (ng/mg-c) after adjustment for sex, age, smoking status, survey episode, surveyed season, BMI, drinking status, and residence area

Target	Observation no.	β	SE	Lower 95% CI	Upper 95% CI	*p*-Value
Total	2,117	0.03	0.01	0.002	0.06	0.04
Males	571	0.02	0.03	–0.04	0.09	0.46
Females	1,546	0.03	0.01	0.002	0.06	0.04

3-PBA, 3-phenoxybenzoic acid; SE, standard error; CI, confidence interval.

Table A2 Relations of insecticide spray use (frequency per week) with log_2-transformed urinary 3-PBA levels (ng/mg-c) after adjustment for sex, age, smoking status, survey episode, surveyed season, BMI, drinking status, residence area, and potential occupational exposure

Target	Observation no.	β	SE	Lower 95% CI	Upper 95% CI	*p*-Value
Total	2,117	0.03	0.01	0.002	0.06	0.03
Males	571	0.02	0.03	–0.04	0.09	0.45
Females	1,546	0.03	0.01	0.002	0.06	0.03

3-PBA, 3-phenoxybenzoic acid; SE, standard error; CI, confidence interval.

Table A3 Relations of insecticide spray use (frequency per week) with log_2-transformed urinary 3-PBA levels (ng/ml) after adjustment for sex, age, smoking status, survey episode, surveyed season, and urinary creatinine level

Target	Observation no.	β	SE	Lower 95% CI	Upper 95% CI	*p*-Value
Total	2,132	0.03	0.01	0.01	0.06	0.01
Males	575	0.03	0.03	–0.03	0.09	0.34
Females	1,557	0.04	0.02	0.01	0.07	0.02

3-PBA, 3-phenoxybenzoic acid; SE, standard error; CI, confidence interval.

Table A4 Relations of insecticide spray use (frequency per week) with log_2-transformed LOD-corrected 3-PBA levels (ng/mg-c) after adjustment for sex, age, smoking status, survey episode, and surveyed season

Target	Observation no.	β	SE	Lower 95% CI	Upper 95% CI	*p*-Value
Total	2,132	0.03	0.01	0.004	0.06	0.02
Males	575	0.03	0.03	–0.03	0.09	0.37
Females	1,557	0.03	0.01	0.003	0.06	0.03

3-PBA, 3-phenoxybenzoic acid; SE, standard error; CI, confidence interval.

Table A5 Relations of the consumption of vegetables and herbal refreshment with log_2-transformed urinary 3-PBA levels (ng/mg-c) after adjustment for sex, age, smoking status, survey episode, and surveyed season

					95% CI		
Dietary intake	Target	Observation no.	β	SE	Lower	Upper	*p*-Value
Vegetables	Total	2,104	0.03	0.03	–0.03	0.09	0.34
	Males	568	0.08	0.07	–0.06	0.23	0.25
	Females	1,536	0.02	0.04	–0.05	0.09	0.65
Herbal refreshment	Total	2,108	–0.06	0.04	–0.14	0.02	0.16
	Males	570	–0.05	0.09	–0.23	0.14	0.61
	Females	1,538	–0.06	0.05	–0.15	0.03	0.20

3-PBA, 3-phenoxybenzoic acid; SE, standard error; CI, confidence interval.

Figure A1 A penalized regression spline of insecticide spray use (frequency/week) on urinary 3-PBA level (ng/mg-c) after adjustment for sex, age, smoking status, survey episode, surveyed season, BMI, drinking status, and residence area

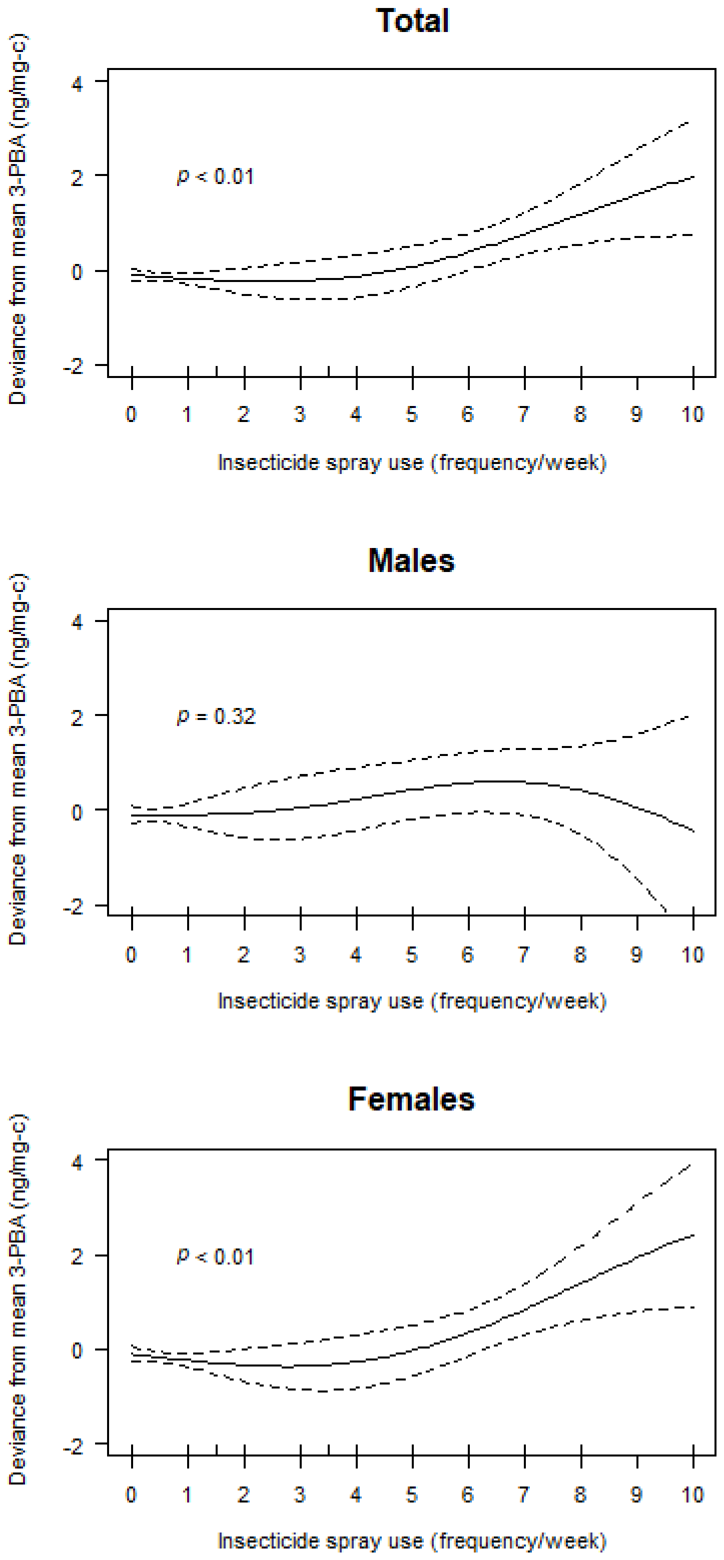

Figure A2 A penalized regression spline of insecticide spray use (frequency/week) on urinary 3-PBA level (ng/mg-c) after adjustment for sex, age, smoking status, survey episode, surveyed season, BMI, drinking status, residence area, and potential occupational exposure

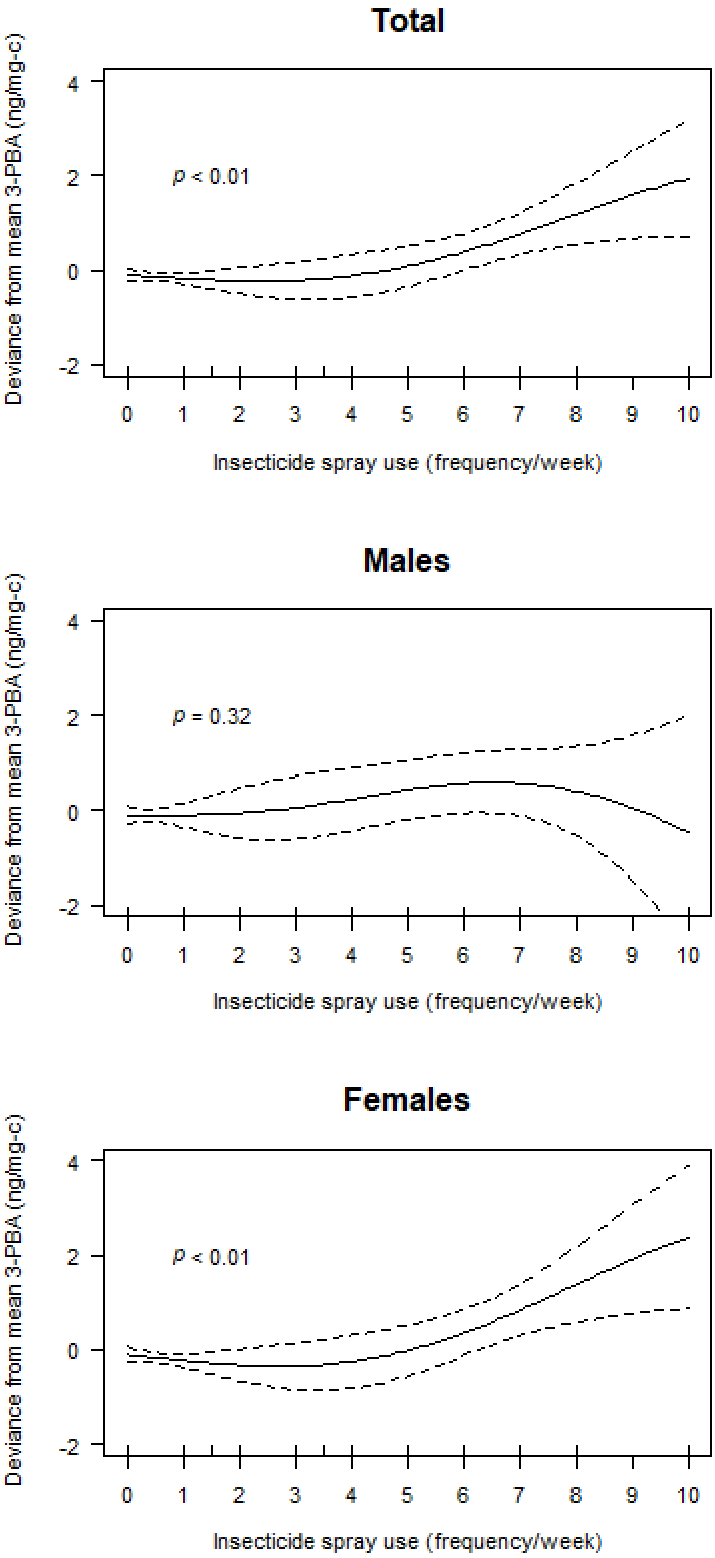

Figure A3 A penalized regression spline of insecticide spray use (frequency/week) on urinary 3-PBA level (ng/ml) after adjustment for sex, age, smoking status, survey episode, surveyed season, and urinary creatinine level

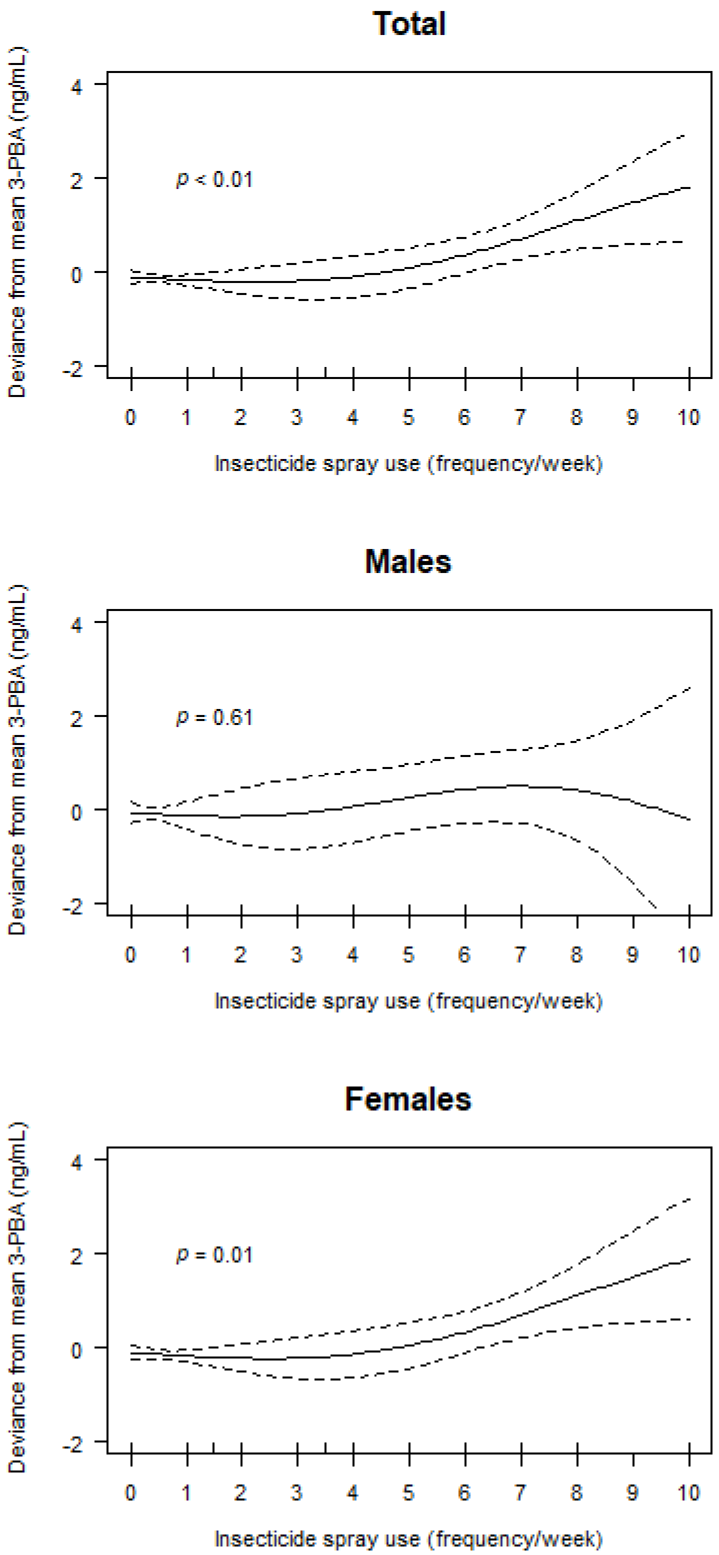

Figure A4 A penalized regression spline of insecticide spray use (frequency/week) on LOD-corrected 3-PBA levels (ng/mg-c) after adjustment for sex, age, smoking status, survey episode, and surveyed season

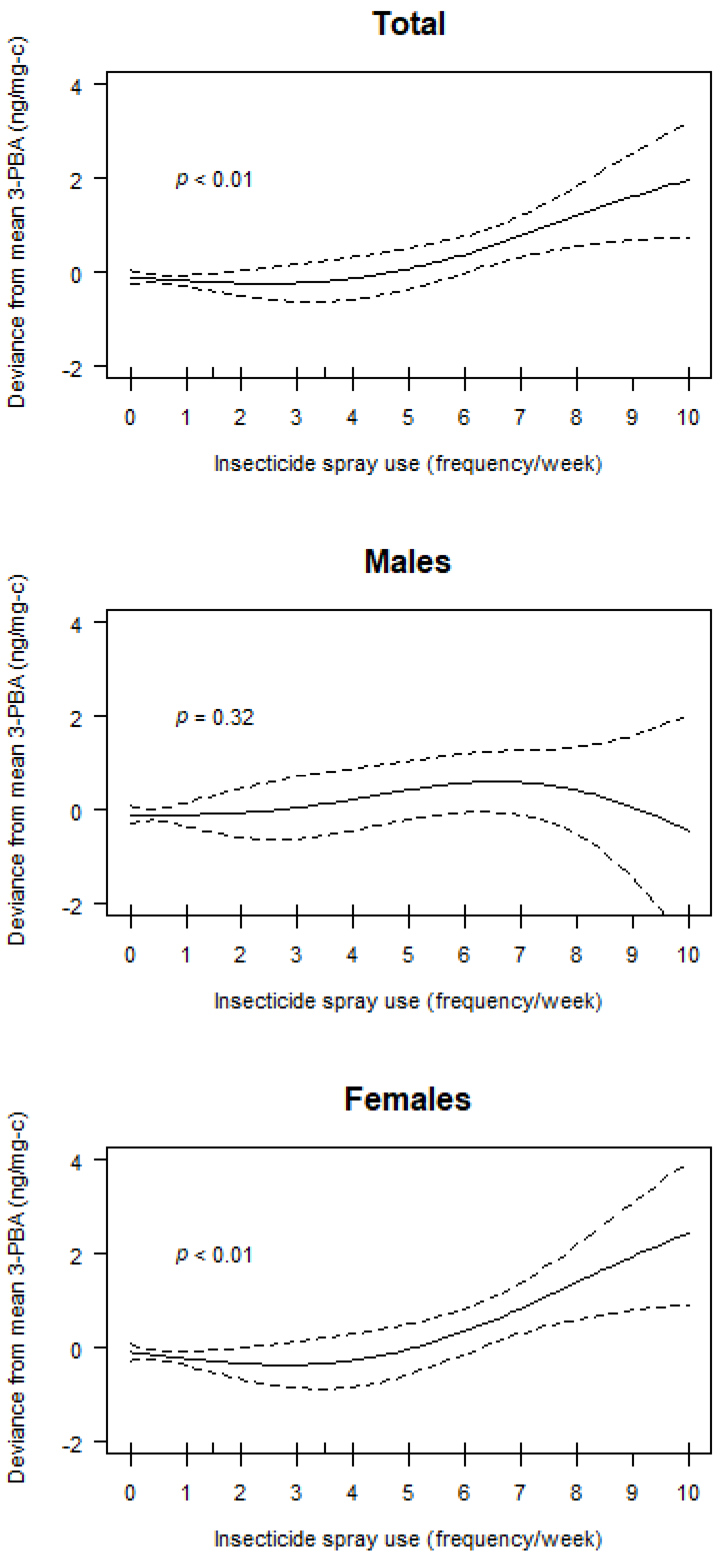

Supplement: Supplementary file 1 — Appendix A [file 41370_2020_276_MOESM1_ESM.pdf]
